# Response to: ‘Nodding syndrome, many questions remain but we can prevent it by eliminating onchocerciasis’

**DOI:** 10.1093/braincomms/fcaa229

**Published:** 2021-01-06

**Authors:** Sam Olum, Peter Scolding, Charlotte Hardy, James Obol, Neil J Scolding

**Affiliations:** 1 Faculty of Medicine, Gulu University, Uganda; 2 Imperial College Healthcare NHS Trust, London, UK; 3 Royal United Hospital, Bath, UK; 4 Institute of Clinical Neurosciences, University of Bristol, Bristol, UK

We thank Dr Gumisiriza *et al.* (in press) for their interest in our review (Olum *et al.*, 2020), and for drawing attention to their two subsequently published 2020 studies exploring the association of onchocerciasis elimination with the incidence of epilepsy in general and nodding syndrome in particular (Gumisiriza *et al.*, 2020*a*, *b*). They conducted epilepsy surveys in northern and in western Uganda and compared their results with historical surveys, respectively, from 2012 and in 1994 (during which interval, community-directed ivermectin treatment and ground larviciding programmes had been completed). In both areas, there was a clear fall in nodding syndrome incidence. Colebunders and colleagues have also now identified nodding syndrome in many other onchocerciasis endemic areas, including Cameroon, the Democratic Republic of Congo and Liberia. The authors draw cause-and-effect conclusions from these and other observations linking epilepsy to onchocerciasis (Colebunders *et al.*, 2017).

These observations are certainly consistent with causality, though the authors stress the absence of a convincing mechanistic explanation: certainly, few if any of Koch’s postulates have been met. As we indicated in our review (Olum *et al.*, 2020), no trace of the organism has been found in CSF or brain autopsy studies (excluding the possibility that the organism might be cultured from CNS tissue). Colebunders and others have very recently added to this literature, showing that even in individuals with epilepsy (not nodding syndrome) and active skin onchocercal infection, neither microfilariae nor parasite DNA were detected in any of the CSF samples by light microscopy or polymerase chain reaction, and no zebrafish injected with the CSF developed seizures (Hotterbeekx *et al.*, 2020). In none of the large animal, non-human primate or rodent experimental onchocerciasis models has epilepsy been reported—and so the fourth postulate, re-isolation, also cannot be met.

An alternative, post-infectious autoimmune relationship to onchocerciasis has (again as we summarized) been suggested by the finding of leiomodin-1 antibodies the sera and CSF of nodding syndrome patients (Johnson *et al.*, 2017)—but only half of nodding syndrome cases appear to have autoantibodies, while a third of healthy controls have the same putatively highly neurotoxic autoantibodies; and autopsy studies do not show the inflammatory changes seen in antibody-mediated encephalitis.

Colebunders and colleagues also emphasize in their studies that, during the interval between surveys (5 years in one study, 34 years in the other), many other changes affecting the population studied will have occurred. The authors successfully controlled for several of these, but others will have included general improvements in public health, the final resolution of internal civil and military conflicts, and the introduction of successful AIDS prevention and treatments, to name but a few. Additionally, widely administered ivermectin will have had therapeutic effects on a significant number of infectious agents other than onchocercal species, as a consequence of its additional antimalarial, antibacterial and antiviral effects (Ōmura and Crump, 2014) (—which are now claimed to include a putative anti-COVID-19 effect! (Chaccour *et al.*, 2020)). Indeed, ivermectin also appears to have anti-seizure activity (Mayer and Horton, 1991). Additionally, nodding syndrome has been observed in areas of Uganda not identified as endemic for onchocerciasis.

That said, any deficiency in absolute proof of a causal link to nodding syndrome by no means proves the absence of a link; and we entirely agree with Gumisiriza, Vieri and Colebunders that onchocerciasis remains a widespread, serious and highly treatable condition. We also agree that further research is necessary and important—and we agree in particular that the need for more studies ‘does not justify inaction while communities and individuals remain seriously affected by this condition’ (Colebunders *et al.*, 2019). Although ivermectin is in fact by no means not entirely free of significant adverse effects (Boussinesq *et al.*, 2003), onchocerciasis elimination programmes do work and should remain a global health priority.

## Data availability

Data sharing is not applicable to this article as no new data were created or analysed.

## Competing interests

The authors report no competing interests.

## References

[fcaa229-B1] Boussinesq M, Gardon J, Gardon-Wendel N, Chippaux JP. Clinical picture, epidemiology and outcome of Loa-associated serious adverse events related to mass ivermectin treatment of onchocerciasis in Cameroon. Filaria J 2003; 2: S4. 14975061 10.1186/1475-2883-2-S1-S4 PMC2147657

[fcaa229-B2] Chaccour C, Abizanda G, Irigoyen-Barrio A, Casellas A, Aldaz A, Martinez-Galan F, et al Nebulized ivermectin for COVID-19 and other respiratory diseases, a proof of concept, dose-ranging study in rats. Sci Rep 2020; 10: 17073. 33051517 10.1038/s41598-020-74084-y PMC7555481

[fcaa229-B3] Colebunders R, Njamnshi AK, van Oijen M, Mukendi D, Kashama JM, Mandro M, et al Onchocerciasis-associated epilepsy: from recent epidemiological and clinical findings to policy implications. Epilepsia Open 2017; 2: 145–52. 29588943 10.1002/epi4.12054 PMC5719844

[fcaa229-B4] Colebunders R, Siewe Fodjo JN, Hopkins A, Hotterbeekx A, Lakwo TL, Kalinga A, et al From river blindness to river epilepsy: implications for onchocerciasis elimination programmes. PLoS Negl Trop Dis 2019; 13: e0007407. 31318857 10.1371/journal.pntd.0007407 PMC6638735

[fcaa229-B5] Gumisiriza N, Kaiser C, Asaba G, Onen H, Mubiru F, Kisembo D, et al Changes in epilepsy burden after onchocerciasis elimination in a hyperendemic focus of western Uganda: a comparison of two population-based, cross-sectional studies. Lancet Infect Dis 2020a; 20: 1315–23. 32598869 10.1016/S1473-3099(20)30122-5

[fcaa229-B6] Gumisiriza N, Mubiru F, Siewe Fodjo JN, Mbonye Kayitale M, Hotterbeekx A, Idro R, et al Prevalence and incidence of nodding syndrome and other forms of epilepsy in onchocerciasis-endemic areas in northern Uganda after the implementation of onchocerciasis control measures. Infect Dis Poverty 2020b; 9: 12. 32114979 10.1186/s40249-020-0628-3 PMC7050130

[fcaa229-B7] Gumisiriza N, Vieri MK, Colebunders R. Nodding syndrome, many questions remain but we can prevent it by eliminating onchocerciasis. Brain Commun in press; 10.1093/braincomms/fcaa228 PMC7811754 33502388

[fcaa229-B8] Hotterbeekx A, Raimon S, Abd-Elfarag G, Carter JY, Sebit W, Suliman A, et al Onchocerca volvulus is not detected in the cerebrospinal fluid of persons with onchocerciasis-associated epilepsy. Int J Infect Dis 2020; 91: 119–23. 31786246 10.1016/j.ijid.2019.11.029 PMC6996151

[fcaa229-B9] Johnson TP, Tyagi R, Lee PR, Lee MH, Johnson KR, Kowalak J, et al Nodding syndrome may be an autoimmune reaction to the parasitic worm Onchocerca volvulus. Sci Transl Med 2017; 9: eaaf6953. doi: 10.1126/scitranslmed.aaf6953. 10.1126/scitranslmed.aaf6953 PMC5434766 28202777

[fcaa229-B10] Mayer TW, Horton ML. Modulation of monomethylhydrazine-induced seizures by ivermectin. Toxicol Lett 1991; 57: 167–73. 1853361 10.1016/0378-4274(91)90143-t

[fcaa229-B11] Olum S, Scolding P, Hardy C, Obol J, Scolding NJ. Nodding syndrome: a concise review. Brain Commun 2020; 2: fcaa037. 32954295 10.1093/braincomms/fcaa037 PMC7425334

[fcaa229-B12] Ōmura S, Crump A. Ivermectin: panacea for resource-poor communities? Trends Parasitol 2014; 30: 445–55. 25130507 10.1016/j.pt.2014.07.005

